# Chromium(III) Glycinate Complex Supplementation Improves the Blood Glucose Level and Attenuates the Tissular Copper to Zinc Ratio in Rats with Mild Hyperglycaemia

**DOI:** 10.1007/s12011-019-01686-7

**Published:** 2019-03-02

**Authors:** Ewelina Król, Zbigniew Krejpcio, Monika Okulicz, Hanna Śmigielska

**Affiliations:** 1 Insitute of Human Nutrition and Dietetics, Poznań University of Life Sciences, ul. Wojska Polskiego 31, 60-624 Poznan, Poland; 2 Department of Animal Physiology and Biochemistry, Poznań University of Life Sciences, ul. Wołyńska 31, 60-624 Poznan, Poland; 3 Department of Natural Science and Quality Assurance, Poznań University of Economics and Business, al. Niepodległości 10, 61-875 Poznan, Poland

**Keywords:** Chromium(III) glycinate complex, Chromium(III) picolinate, Supplementation, Hyperglycaemia, Diabetic rats

## Abstract

The aim of the study was to evaluate the hypoglycaemic potential of supplementary Cr in the form of chromium(III) glycinate (CrGly) in the diabetic model of rats. The experiment was conducted on 40 male Wistar rats, of which 30 were made diabetic by injection of a single dose of streptozotocin (55 mg/kg b.m.), while the remaining 10 rats served as the healthy control. After inducing hyperglycaemia, 2 groups of diabetic rats (10 rats each) were supplemented with Cr either as CrGly or chromium(III) picolinate (CrPic) given orally at a dose of 10 mg/kg diet (about 0.75 mg Cr/kg b.m.) with adequate AIN-93M diet for 7 weeks. At the termination of experiment, all animals were sacrificed to collect blood and internal organs for biochemical assays. Blood biochemical indices and tissular trace element contents (Fe, Zn, Cu, Cr) were measured and compared with the values of the untreated groups. It was found that CrGly significantly decreased blood glucose, total cholesterol, HDL cholesterol and triacylglycerol levels more efficiently than CrPic. Furthermore, both Cr compounds normalized disturbed the serum, renal and cardiac molar Cu/Zn ratio, as well as restored the kidney Zn and Cu levels in rats with hyperglycaemia. Supplementary Cr did not increase the tissular Cr levels in diabetic rats. The study confirmed the hypoglycaemic potential of CrGly in the diabetic model of rats.

## Introduction

Diabetes is a metabolic disease causing glucose homeostasis disorders. It progresses over time. According to the WHO estimates, in 2014, there were 422 million people aged over 18 years with diabetes worldwide [1]. Therefore, dietary supplements that could improve disturbed carbohydrates balance are very popular, especially in the USA. Recently, the analysis of the NHANES study data and the filtering the database for the required covariates (sex, ethnicity, socioeconomic status, body mass index, diabetes diagnosis, supplement usage and laboratory HbA1c values) showed that 58.3% of adults had consumed a dietary supplement in the previous month. Among them, 28.8% reported consuming a dietary supplement that contained chromium. The authors concluded that the odds ratio of the incidence of type 2 diabetes in the people who consumed chromium-containing supplements within the previous 30 days was lower than in nonusers [2].

Dietary Cr(III) supplementation is generally considered as safe, but in some cases, it may lead to the accumulation of this element in the kidneys [3]. Therefore, Cr(III) supplementation is not recommended for people with kidney or liver disease. Moreover, high doses of Cr(III) supplements affect the homeostasis of other elements.

Although the use of chromium(III) supplements in diabetes management is controversial, researchers are still looking for factors that could influence its efficacy. The most popular form of Cr(III) on dietary supplements market are picolinate, brewer’s yeast enriched with chromium, niacin-bound Cr(III) and chloride. Generally, the efficiency of Cr(III) supplementation depends on the bioavailability of Cr(III), which is still very low ranging from 0.5% (chromium (III) chloride) to 10% (brewer’s yeast) [4]. Recently, these results were verified in rats and on humans [5]. The authors found that among the Cr(III) supplements user analysis (Cr-picolinate, Cr-nicotinate, Cr-phenylalaninate, Cr-proprionate, or Cr-chloride), 7 days after oral administration of ^51^Cr to rats the apparent absorption was low, ranging from 0.04 to 0.24%. These values were higher in three volunteers who took 400 μg Cr, where the highest value (0.95%) was observed for Cr(D-Phen)_3._

The problem of low bioavailability of chromium could be solved by finding non-toxic ligands facilitating its absorption in animal and humans. In the last decade, many amino acid ligands have been synthetized and tested, e.g. propionate, malate, acetate, histidinate, d-phenylalanine, glycinate and methionine [6–11].

Earlier studies showed that the trinuclear oxo-centered Cr carboxylate structure (as in the chromium(III)–glycine complex) was a key element that determined high bioavailability and biological activity of the Cr(III) compound. In contrast to chromium(III) picolinate (CrPic), CrGly is well soluble in water [12, 13].

In an earlier study, the effect of synthetic Cr(III) complexes (acetate, chloride, glycinate, histidinate, lactate and propionate) on insulin binding and signal transduction was investigated in vitro on three experimental models: isolated rat liver membranes and cultured mouse C2C12 myoblasts and 3T3-L1 preadipocytes [14]. In the absence of insulin, two Cr(III) complexes, i.e. chromium glycinate (CrGly) and acetate (CrAc), slightly elevated the phosphorylation at tyrosine IRS-1. This suggested that in vivo these complexes might significantly enhance inulin signaling inside cells, especially at low levels of insulin in the blood.

The aim of this study was to evaluate the hypoglycaemic potential of the supplementary Cr(III) glycinate (CrGly) complex in comparison with Cr(III) picolinate in mild diabetic model of rats.

## Material and Methods

### Animals

Male Wistar rats (*n* = 40, 6 weeks old, mean initial body weight 175 g) were purchased from the Licensed Laboratory Animals Breeding Center at the Poznań University of Medical Sciences (Poznań, Poland). Throughout the experiment, the rats were kept at animal care facility under controlled temperature (21 ± 2 °C) and humidity (55–60%) with artificial 12 h/12 h day/night cycle. After 7 days of adaptation, the animals were divided into four groups: C—the control group, D—the diabetic control group, D+CrGly—diabetic rats fed a diet supplemented with CrGly at the dose of 10 mg Cr/kg/diet and D+CrPic—diabetic rats fed a diet supplemented with chromium(III) picolinate at a dose of 10 mg Cr/kg/diet.

### Diets

For 7 weeks, the rats were fed semi-synthetic diets composed according to the American Institute of Nutrition (AIN-93M) recommendations [15]. The diets consisted of casein (14%), soybean oil (4%), wheat starch (62.32%), sucrose (10%), potato starch (5%), l-cysteine (0.3%), vitamin mix AIN-93M (1%) and mineral mix AIN-93M (3.5%). Two mineral mixtures were enriched with either CrGly or CrPic to a level of 10 mg elemental Cr/kg diet. The chemical composition of the experimental diets is shown in Table 1. During the entire experimental period, the animals had free access to food and distilled water. The food intake was monitored daily. The body mass gain was monitored weekly.

**Table 1:** The chemical composition of diets used in experiment

Ingredient	Experimental diets			
	C	D	D+CrGly	D+CrPic
Protein	12.93 ± 0.07	12.68 ± 0.38	12.75 ± 0.05	12.21 ± 0.32
Fat	4.92 ± 0.04	4.80 ± 0.10	4.96 ± 0.04	4.86 ± 0.18
Carbohydrates	70.65 ± 0.09	71.10 ± 0.04	70.78 ± 0.07	71.77 ± 0.26
Ash (%)	2.92 ± 0.16	2.81 ± 0.17	2.64 ± 0.21	2.75 ± 0.04
Fe (mg/kg)	48.65 ± 4.38	46.54 ± 5.81	47.19 ± 3.70	49.45 ± 3.21
Zn (mg/kg)	37.56 ± 4.56	38.99 ± 2.45	37.77 ± 4.76	39.76 ± 6.34
Cu (mg/kg)	6.54 ± 2.34	6.43 ± 3.48	5.99 ± 1.23	6.11 ± 1.41
Cr* (mg/kg)	1.34 ± 0.32	1.44 ± 0.24	11.75 ± 0.45	11.90 ± 0.53

*Cr(III) was added to mineral mix in the forms as follows: D+CrGly—chromium(III) glycinate; D+CrPic—chromium(III) picolinate

### Induction of Diabetes

After the acclimatisation period, the rats were intraperitoneally injected with STZ (55 mg/kg body mass in citric buffer, pH = 4.4, Sigma-Aldrich (Saint Louis, MO, USA)) to induce hyperglycaemia and diabetes. The rats from the control group were injected only with the carrier (citric buffer, pH 4.4). Three days after the injection, blood drops were taken from the tail vein and placed on test strips to measure the blood glucose concentration (Optium Medisense glucometer, Abbott Co., USA). After the injection of STZ, all the animals exhibited elevated fasting blood glucose levels (> 9 mmol/l) and were classified and named as “mild diabetic” (D).

### Test Chemicals

Chromium(III) complex with glycine (CrGly) in the form of nitrate salt (chemical formula [Cr_3_O(NH_2_CH_2_CO_2_)_6_(H_2_O)_3_] + NO_3_·H_2_O, the content of Cr 25.7%), as an analogue of chromium(III) propionate complex (Cr3, chemical formula [Cr_3_O(O_2_CCH_2_CH_3_)_6_(H_2_O)3]^+^ NO_3_) was synthesized in the laboratory of the Department of Product Ecology, Poznan University of Economics and Business, according to the method described previously [16].

Chromium(III) picolinate (CrPic) (Nutrition 21, Inc. NY, USA) was kindly provided by Professor Kazim Sahin from Firat University (Elazig, Turkey).

The content of elemental Cr in the compounds was determined by the AAS method (spectrometer AAS-3 with BC correction, Zeiss, Germany).

### Data Collection

After 16 h fasting, the rats were anesthesized with an intraperitoneal thiopental injection (40 mg/kg b.m.) and dissected to collect blood from the aorta. The inner organs (liver, kidneys, heart, spleen, pancreas, testes) were removed. Their blood was collected into test tubes containing EDTA. Next, it was centrifuged (3500×*g* for 10 min, 4 °C) and stored at − 20 °C for biochemical tests.

Internal organs and tissues were washed in saline, weighed and stored at − 20 °C until analysis. All the procedures applied in this study were accepted by the Animal Bioethics Committee of Poznań, Poland (Approval no. 47/2010).

## Laboratory Analyses

### Blood Biochemistry

The blood serum indices were measured with standard methods using an Olympus AU 560 analyser (Tokyo, Japan). In particular, the serum glucose level was measured with the hexokinase method [17]. The serum total, LDL, HDL cholesterol and triacylglycerol concentrations were measured with colorimetric methods [18–20]. The insulin concentration was determined with the RIA method using a rat-specific kit (RI-13K, Millipore Corporation, St. Charles, MO, USA). The serum GST, TBARS and C-reactive protein concentrations were measured with rat-specific kits (Cayman Chemical; 1180 East Ellsworth Road, Ann Arbor, Michigan, USA; Wuhan EIAab Science Co., Biopark, Optics Valley, Wuhan, China).

The ALT and AST enzyme activities were measured with the kinetic methods. The urea concentration was determined by the kinetic method using urease and glutamine dehydrogenase. The total protein concentration was measured with the colorimetric method, using Cu^+2^ ions. The creatinine concentration was measured with the Jaffe’s kinetic method using picric acid [21–23].

Insulin resistance was characterized with the homeostasis model assessment (HOMA) indices adjusted to the rodent model [24, 25].

$$ \mathrm{HOMA}-\mathrm{IR}=\left(\mathrm{Fasting}\ \mathrm{Glucose}\ \left[\mathrm{mmol}/\mathrm{L}\right]\times \mathrm{Fasting}\ \mathrm{Insulin}\ \left[\mathrm{mIU}/\mathrm{L}\right]\right)/22.5 $$

### Microelements Determination

The rats’ tissues were digested in 65% (*w*/*w*) spectra pure HNO_3_ (Suprapur, Merck KGaA, Darmstadt, Germany) in the Microwave Digestion System (Mars-5, CEM Corp., Matthews NC, USA). Then, the concentrations of Fe, Zn and Cu in the mineral solutions were measured with the F-AAS method (AAS-3 spectrometer with BC, Zeiss, Germany). The content of Cr was measured with the graphite furnace AAS method (AA EA 5 spectrometer with BC, Jenoptic, Germany).

The accuracy of quantitative measurements of Fe, Zn, Cu and Cr was assured by simultaneous analysis of certified reference materials (bovine liver NIST® SRM® 1577c and human serum HN2612 (Randox Laboratories, Crumlin, UK)). The mean recoveries of the reference material for tissues were as follows: Fe—98%, Zn—95%, Cu—102%, Cr—103% and for the serum Fe—105%, Zn—101%, Cu—99%.

The limit of detection (LOD) for the method of Cr determination was calculated by the equation LOD = 3*s*/*m* (*s*—standard deviation of the measurements of the blank; *m*—the slope of the calibration curve). Precision for the method was expressed as coefficient of variance (CV). These validation parameters were, respectively, LOD = 0.32 μg/L and CV = 6.34%.

### Statistical Analysis

All the results were presented as means values ± standard deviation. One-way analysis of variance (ANOVA) with post-hoc Tukey’s test was used to determine statistically significant differences in the means values for each variable. The associations between variables were checked with the Pearson’s correlation coefficient. The mean values were considered statistically different at *p* < 0.05. The STATISTICA (ver. 10.0) program (StatSoft, Inc., Tulsa, USA) was used for all calculations.

## Results

Table 2 shows the effects of chronic hyperglycaemia and supplementary Cr(III) compounds on overall nutritional indices in rats. Generally, hyperglycaemia induced by STZ injection (partial pancreatic cell damage) and inefficient glucose utilisation by cells brought about metabolic disorders. Food intake only slightly (insignificantly) increased (*p* > 0.05) to compensate for reduced energy availability; however, at the end of experiment, no statistically significant differences in body mass gain and body mass/body length ratio between all experimental groups were observed. Dietary Cr intake, as expected, was 7–8-fold higher in the Cr treated diabetic groups (D+CrGly and D+CrPic) due to supplementation. Diabetic rats supplemented with CrGly consumed markedly less food by 12% on daily basis, as compared with diabetic control rats (*p* < 0.05). The feeding efficacy ratio (FER) as well as the absolute and relative internal organ masses were not statistically different among the experimental groups (*p* > 0.05).

**Table 2:** Effect of different chromium(III) complexes on overall growth indices in diabetic rats

Index		Experimental group			
		C	D	D+CrGly	D+CrPic
Food intake (g/day)		20.29 ± 1.05AB	22.90 ± 4.46B	20.05 ± 0.87A	20.80 ± 1.46AB
Fe intake (g/day)		0.99 ± 0.05	1.07 ± 0.21	0.95 ± 0.04	1.03 ± 0.07
Zn intake (g/day)		0.76 ± 0.08	0.91 ± 0.26	0.72 ± 0.07	0.81 ± 0.08
Cu intake (g/day)		0.13 ± 0.01AB	0.15 ± 0.03B	0.12 ± 0.01A	0.13 ± 0.01AB
Cr intake (g/day)		0.027 ± 0.001A	0.033 ± 0.006A	0.236 ± 0.010B	0.248 ± 0.017B
Body mass gain (g/7 weeks)		170.40 ± 26.20	155.00 ± 35.90	171.22 ± 10.52	159.11 ± 26.47
Body mass/body length ratio (g/cm)		14.35 ± 0.72	13.49 ± 0.92	14.06 ± 0.46	13.61 ± 1.16
FER (g/100 g feed)		18.66 ± 1.87	16.09 ± 5.93	19.36 ± 1.51	17.14 ± 2.97
Liver	(g)	10.89 ± 0.58	12.02 ± 2.86	11.34 ± 1.23	11.72 ± 1.20
	(% b.m.)	3.08 ± 0.13	3.38 ± 0.80	3.19 ± 0.35	3.29 ± 0.34
Kidneys	(g)	2.35 ± 0.16	2.68 ± 0.58	2.43 ± 0.21	2.42 ± 0.24
	(% b.m.)	0.66 ± 0.05	0.75 ± 0.16	0.68 ± 0.06	0.68 ± 0.07
Spleen	(g)	0.62 ± 0.05	0.60 ± 0.08	0.64 ± 0.04	0.61 ± 0.05
	(% b.m.)	0.17 ± 0.02	0.17 ± 0.02	0.18 ± 0.01	0.17 ± 0.01
Heart	(g)	0.97 ± 0.05	0.91 ± 0.10	0.99 ± 0.06	0.95 ± 0.14
	(% b.m.)	0.27 ± 0.02	0.26 ± 0.03	0.28 ± 0.02	0.27 ± 0.04
Testes	(g)	3.36 ± 0.14	3.46 ± 0.19	3.53 ± 0.13	3.26 ± 0.32
	(% b.m.)	0.94 ± 0.04	0.97 ± 0.05	0.99 ± 0.04	0.92 ± 0.09
Pancreas	(g)	1.09 ± 0.12	1.12 ± 0.09	1.06 ± 0.19	1.16 ± 0.12
	(% b.m.)	0.31 ± 0.03	0.31 ± 0.02	0.30 ± 0.05	0.33 ± 0.03

Data are expressed as mean ± standard deviation (SD). Values in the row without common uppercase letters differ significantly at *p* < 0.05

*FER* food efficacy ratio, *b.m.* body mass

Table 3 shows the effects of hyperglycaemia and supplementary Cr(III) compounds (CrGly and CrPic) on the diabetic rats’ glucolipid indices. A single STZ injection at a dose of 55 mg/kg b.m., as expected, significantly increased (*p* < 0.05) the rats’ blood glucose level over 9 mmol/L, inducing hyperglycaemia in rats. Supplementary Cr in the form of CrGly administered at a dose of 10 mg Cr/kg diet (ca. 0.75 mg Cr/kg b.m.) restored the blood glucose level almost to the healthy rats’ level, whereas CrPic only slightly decreased hyperglycaemia in the diabetic rats. The blood insulin levels varied considerably in experimental animals, especially after STZ injection, so no statistical differences were detected between the groups. Also, the calculated insulin resistance index (HOMA-IR) was very scattered in each group. Therefore, there was no significant difference between healthy, Cr-treated and untreated diabetic groups. Chronic hyperglycaemia disorders overall metabolism, especially glucose, lipid, microelement metabolism, as well as in bodily redox balance, which are responsible for further diabetic complications. In the experiment, the diabetic untreated rats had higher blood total cholesterol (*p* < 0.05) and HDL cholesterol levels and slightly elevated serum triacylglycerols levels. Supplementary CrGly normalized the blood those lipid indices to the level noted in the healthy control rats (*p* < 0.05).

**Table 3:** Effect of different chromium(III) complex supplementations on serum biochemical indices of diabetic rats

Parameter	Experimental group			
	C	D	D+CrGly	D+CrPic
Glucose (mmol/L)	7.05 ± 0.30A	9.53 ± 0.96B	7.53 ± 0.92A	8.07 ± 0.94AB
Insulin (μg/L)	1.82 ± 0.38	0.96 ± 0.54	0.62 ± 0.25	1.52 ± 1.01
HOMA-IR index	2.27 ± 0.31	1.53 ± 0.81	0.84 ± 0.43	2.18 ± 1.46
Total cholesterol (mmol/L)	2.18 ± 0.20A	2.56 ± 0.36B	2.23 ± 0.28A	2.33 ± 0.30AB
HDL cholesterol (mmol/L)	1.58 ± 0.10A	1.94 ± 0.28B	1.66 ± 0.27A	1.73 ± 0.24AB
LDL cholesterol (mmol/L)	0.21 ± 0.20	0.31 ± 0.16	0.25 ± 0.13	0.20 ± 0.09
Triacylglycerols (mmol/L)	0.91 ± 0.16AB	1.34 ± 0.26B	0.85 ± 0.51A	1.10 ± 0.50AB
ALT (U/L)	16.00 ± 2.24	22.22 ± 7.12	18.22 ± 4.55	18.67 ± 4.44
AST (U/L)	75.00 ± 9.63	95.78 ± 22.21	77.78 ± 11.95	82.13 ± 11.17
Creatinine (μmol/L)	5.57 ± 0.74	6.83 ± 2.77	5.30 ± 0.49	5.57 ± 0.88
Urea (mmol/L)	32.20 ± 4.46	41.00 ± 16.04	31.78 ± 2.91	33.44 ± 5.45
GST (nmol/min)	2.29 ± 0.74	3.06 ± 0.39	2.98 ± 0.31	2.51 ± 1.14
TBARS (μM/L)	27.43 ± 6.65	34.70 ± 9.06	27.95 ± 5.16	33.71 ± 3.28
CRP (μg/L)	0.08 ± 0.01	0.11 ± 0.04	0.11 ± 0.02	0.11 ± 0.03

Data are expressed as mean ± standard deviation. Values in the row without common uppercase letters differ significantly at *p* < 0.05

*HOMA-IR* Homeostasis Model Assessment for Insulin Resistance, *ALT* alanine aminotransferase activity, *AST* aspartate aminotransferase activity, *GST* glutathione *S*-transferase, *TBARS* thiobarbituric acid reactive substances, *CRP* C reactive protein

Hyperglycaemia induced by the STZ injection only slightly increased the toxicity indices (ALT activity, AST activity, creatinine and urea), but these changes were not statistically different between the groups. Supplementary Cr (both CrGly and CrPic) did not affect these parameters in blood of rats.

Neither hyperglycaemia or supplementary Cr(III) complexes had significant influence on serum GST, TBARS and CRP levels.

Table 4 shows the effects of hyperglycaemia and supplementary Cr on the mineral status in the diabetic rats. In our study, the induction of diabetes with the STZ injection at a relatively low dose did not affect the serum Fe, Zn and Cu levels, but it increased the serum Cu/Zn molar ratio by 9.5%. This change was further normalized by supplementary Cr (both CrGly and CrPic, 10 mg Cr/kg of diet, about 0.75 mg Cr/kg b.m.).

**Table 4:** The contents of Fe, Zn and Cu in serum and tissues and Cr in liver and kidney of rats

Index	Experimental group			
	C	D	D+Cr(III)Gly	D+Cr(III)Pic
Serum (μmol/L)				
Fe	26.05 ± 3.91	29.26 ± 6.80	24.32 ± 5.25	21.98 ± 3.58
Zn	18.47 ± 1.38	18.61 ± 3.07	18.70 ± 3.36	18.98 ± 2.16
Cu	15.49 ± 0.75	17.11 ± 2.39	15.80 ± 1.11	15.09 ± 1.04
Molar Cu/Zn ratio	0.84 ± 0.08A	0.92 ± 0.09B	0.79 ± 0.11A	0.81 ± 0.12A
Liver (μg/g d.m.)				
Fe	215.24 ± 21.84	257.51 ± 26.72	277.94 ± 48.27	248.87 ± 43.16
Zn	100.71 ± 17.92	99.63 ± 10.25	99.53 ± 11.15	94.99 ± 9.17
Cu	20.41 ± 3.08	19.65 ± 1.90	18.85 ± 2.25	19.75 ± 1.53
Molar Cu/Zn ratio	0.211 ± 0.022	0.203 ± 0.009	0.203 ± 0.019	0.215 ± 0.013
Cr	0.761 ± 0.132	0.663 ± 0.242	0.727 ± 0.327	0.734 ± 0.245
Kidney (μg/g d.m.)				
Fe	255.80 ± 33.10	232.63 ± 28.52	240.25 ± 37.61	236.29 ± 35.86
Zn	112.38 ± 7.36B	90.18 ± 20.26A	111.86 ± 14.11B	108.78 ± 5.10B
Cu	48.48 ± 5.80A	113.99 ± 48.90B	95.24 ± 6.12A	58.54 ± 8.54A
Molar Cu/Zn ratio	0.427 ± 0.044A	1.366 ± 0.750B	0.509 ± 0.039A	0.555 ± 0.083A
Cr	0.992 ± 0.236	0.890 ± 0.239	0.956 ± 0.239	0.994 ± 0.387
Heart (μg/g d.m.)				
Fe	318.52 ± 22.25	321.13 ± 34.65	313.03 ± 37.92	310.11 ± 36.30
Zn	68.79 ± 6.11A	58.57 ± 7.15A	61.13 ± 6.15AB	65.42 ± 11.24AB
Cu	20.41 ± 3.08	19.65 ± 1.90	18.85 ± 2.25	19.75 ± 1.53
Molar Cu/Zn ratio	0.338 ± 0.031A	0.472 ± 0.079B	0.405 ± 0.056A	0.345 ± 0.034A
Spleen (μg/g d.m.)				
Fe	2749.55 ± 472.87	3206.80 ± 454.50	3083.29 ± 479.73	3026.95 ± 461.85
Zn	61.62 ± 7.87	83.79 ± 9.14	77.42 ± 3.77	65.42 ± 11.24
Cu	48.48 ± 5.80	113.99 ± 48.90	95.24 ± 6.12	58.54 ± 8.54
Molar Cu/Zn ratio	0.267 ± 0.073	0.257 ± 0.091	0.226 ± 0.088	0.258 ± 0.099

Data are expressed as mean ± standard deviation (SD). Values in the row without common uppercase letters differ significantly at *p* < 0.05

The liver Fe, Zn and Cu contents were not different in the experimental groups. Also, the liver Cr levels were not affected by the supplementary Cr. It confirmed earlier observations that this element was poorly accumulated in the liver.

Hyperglycaemia and supplementary Cr did not change the kidney Fe contents in rats. By contrast, the liver Zn level decreased by 20% in diabetic untreated rats, whereas supplementary Cr (both CrGly and CrPic) ameliorated these changes in the diabetic rats. The liver Cu content increased by 135% in the untreated diabetic group, whereas supplementary Cr (both CrGly and CrPic) was able to correct this change almost to the level of the healthy control group. Changes in the renal Zn and Cu levels brought about shifts in the kidney Cu/Zn molar ratio. The index increased by about 200% in the diabetic group, whereas supplementary Cr (both forms) corrected this ratio to the level noted in the healthy control group. Neither hyperglycaemia nor supplementary Cr affected the rats’ hepatic and renal Cr levels.

The heart Fe and Cu levels were not statistically different in any of the experimental groups. The heart Zn content decreased by 15% in the diabetic untreated rats and supplementary Cr did not fully correct this shift to the level of the healthy control group. Furthermore, the heart Cu/Zn molar ratio increased by 40% in the hyperglycemic untreated rats. Supplementary Cr ameliorated this change nearly to the level noted in the healthy control rats.

The spleen Fe, Zn, Cu and Cr levels were not statistically different in the experimental groups.

Table 5 shows the significant correlation coefficients between serum biochemical indices and tissular mineral levels in diabetic rats. The positive associations were found between serum TBARS and serum insulin (*r* = 0.576), HOMA-IR (*r* = 0.617) and triacylglycerols (*r* = 0.649) levels. The serum glucose correlated with renal Cu level (*r* = 0.622) and renal Cu/Zn ratio (*r* = 0.575), while serum triacylglycerols with splenic Zn level (*r* = 0.472).

**Table 5:** Significant correlations between serum biochemical indices and tissular mineral levels in diabetic rats

Correlation	Pearson’s correlation coefficient	
	*r*	*p*
Insulin—TBARS	0.576	0.020
HOMA-IR—TBARS	0.617	0.014
Triacylglycerols—TBARS	0.649	0.009
Glucose—renal Cu	0.622	0.001
Glucose—renal Cu/Zn	0.575	0.004
Triacylglycerols—splenic Zn	0.472	0.017

*HOMA-IR* Homeostasis Model Assessment for Insulin Resistance, *TBARS* thiobarbituric acid reactive substances, *p* probability value for correlation

## Discussion

The first study on the beneficial effect of Cr(III) on the regulation of carbohydrate metabolism was published in the late 1950s when Schwartz and Mertz et al. [26] found that Cr(III) appeared to be an essential element in glucose metabolism. Since then, there have been numerous studies on animals [6–8, 27, 28] and humans [29–31], which analysed the role of this element in carbohydrate and lipid metabolism. Many studies on animal-resistant and diabetic animals (mice, rats) showed the therapeutic potential of various supplementary Cr(III) compounds, which regulated disordered carbohydrate and lipid metabolism in diabetes. On the other hand, clinical trials conducted on healthy and diabetic patients supplemented with various forms of Cr (both organic and inorganic), given in a wide range of doses and duration, gave highly variable or contradictory results. Yin and Phung [32] reviewed existing reports comprising 14 well-controlled randomized or interventional studies. They concluded that among different Cr(III) compounds only brewer’s yeast Cr improved fasting plasma glucose levels, but it did not affect the glycated hemoglobin level in DM2 patients. The ineffectiveness of supplementary Cr in managing diabetes was widely discussed by Costello et al. [33]. According to Vincent [34], the beneficial effect of Cr(III) supplements on humans was not supported by evidence based research and the discrepancies between animal models and clinical trials resulted from the Cr dosages used in those trails. Supranutritional or pharmacological dosages were usually applied in studies on animals but they did not correspond to those used by human subjects. Supplementary Cr(III) usually exerts its hypoglycaemic potential only when given in very high dosages (> 1 mg Cr/day), but the risk of side effects and toxicity increases considerably.

Diabetes mellitus is a common non-communicable disease with increasing morbidity rates among many populations worldwide. There are different types of diabetes known among humans, but the type 2 diabetes mellitus has the highest prevalence. Researches describe several animal models of insulin resistance and diabetes. One of the most useful and simple method to induce hyperglycaemia in small rodents (mice or rats) relies on pharmacological injection of chemicals, e.g. alloxan or streptozotocin (STZ) that partly destroy pancreatic β cell function. According to different authors, depending on the injected dose of STZ, different types of diabetes (type 1 or type 2) can be mimicked in experimental animals. For example, a single injection of STZ to rats at a dose of > 60 mg/kg b.m. can lead to drastic destruction of β cells; thus, insulin secretion declines resulting in significant chronic hyperglycaemia. In turn, a smaller injected dose of STZ (40–55 mg/kg b.m.) and feeding a normal diet leads to the onset of symptoms of type 2 diabetes. This type of diabetes can also be induced by injecting a lower dose of STZ (35 mg/kg b.m.) preceded by feeding a high-fat diet.

In our study, the hypoglycemic potential of newly synthesized CrGly and CrPic was compared in tests on rats with “moderate” hyperglycaemia induced by a single injection of STZ (55 mg/kg b.m.). In consequence, the blood glucose level in rats that were administered STZ increased, but the changes were relatively mild, as a consequence of the adding of the mentioned chromium compounds to the diet. Simultaneously, the concentrations of serum insulin levels decreased; total and HDL cholesterol levels and triacylglycerols level increased moderately. During the experiment, supplementary Cr (10 mg Cr/kg diet, about 0.75 mg Cr/kg b.m.) partially ameliorated the disordered glucose metabolism and related lipid indices. The hypoglycaemic effect of CrGly was more pronounced than of CrPic, at the same dietary doses.

The reason for the stronger effect of CrGly is unknown, but it may be caused by better stability, solubility in water (e.g. CrPic is poorly soluble in water), higher rate of intestinal absorption, or other factors. The mechanisms of the CrPic hyperglycaemia-correcting action have been proposed by various researchers. For example, Chen et al. [35] and Kandadi et al. [36] suggested that the reversal of insulin resistance by CrPic may be mediated by the inhibition of c-Jun NH2-terminal kinase (JNK) activation. Pattar et al. [37] reported that CrPic can affect cholesterol homeostasis and lower the blood glucose level by altering the plasma membrane composition of cholesterol in fat and muscle cells. CrPic has been shown to enhance glucose transporters (GLUT-4) translocation. Hyperinsulinemia is another aspect that needs to be taken into account when describing factors affecting the CrPic action on cholesterol metabolism. Hoffman et al. [38] found that CrPic improved insulin action in hyperinsulinemia condition and prevented an increase in membrane cholesterol level. This mechanism compromised F-actin integrity, which is necessary for GLUT-4 translocation regulated by insulin.

Vincent [39] concluded that Cr given at pharmacological doses appeared to increase insulin sensitivity in diabetic and insulin-resistant animals. The mechanism of its action may involve insulin-sensitive movement of Cr to tissues. This suggests that Cr could act as a second messenger and transmitting signals from the receptor to target cells.

In our study, CrGly was shown to have the hypoglycemic potential. This compound was previously studied in the Sahin’s laboratory [40] using high-fat fed rats. It was found that CrGly supplemented in water at doses of 40 and 80 μg/kg b.m./day decreased the blood glucose/insulin ratio, restored depleted tissue Cr concentrations and alleviated memory acquisition in insulin-resistant rats.

Chromium in its trivalent form has the potential to affect the status of trace elements due to possibility of its interactions, especially with Fe. It has further consequences, like depression of Fe uptake and deficiency, shift in the redox balance (Fe, Zn and Cu), which is disturbed in hyperglycaemia.

Cr(III) compounds can interact with Fe, as these two elements use the same transport protein–transferrin. They may exhibit competitive activity [41, 42]. Quarles et al. [42] investigated the competitive activity of Fe^+3^, Cr^+3^ and Ni^+2^ binding to transferrin at different physiological iron/transferrin concentration ratios. These ratios corresponded to different stages of the iron status; normal, iron-deficient, iron-deficient from chronic disease, iron-deficient from inflammation and iron-overload. It was found that the binding of Cr^+3^ by transferrin was reduced when the amount of Fe^+3^ was in extreme excess (more than 50% Fe^+3^-loaded) [42].

In our study, neither supplementary CrGly nor CrPic (about 0.75 mg Cr/kg b.m.) influenced tissular (serum, liver, kidney, heart and spleen) Fe levels, probably due to relatively low Cr load. Similarly, the Sahin’s team [43] performed a study were diabetic rats were supplemented with Cr(III) histidinate complex (CrHis) at low doses (110 μg/kg b.m.) for 10 weeks and did not observed significant changes in tissular Fe levels in those animals.

The interaction of Cr with Fe in vivo occurs or not, depending on the experimental conditions, mainly on the dose of Cr, duration of treatment and metabolic conditions (health, disease). In our previous trial [6], supplementary Cr propionate complex (also known as Cr3), given in doses of 1 and 5 mg/kg b.m. for 8 weeks, decreased the kidney Fe concentration in insulin-resistant rats, whereas in the other trail [27], the same doses of the compound normalized the increased liver Fe content in high-fat diet/STZ injected diabetic rats. Vincent’s group [44] also observed a decreased kidney Fe level in obese type 2 diabetic rats supplemented with 1000 μg Cr/kg b.m.) in the form of Cr3. In another study, the same team [45] found that CrPic and Cr3, given at doses of 1 mg/kg b.m. for 12 weeks, markedly decreased the hepatic and splenic Fe content but had no influence on the renal and heart levels in obese rats. In a recent study by Staniek and Wójciak [46], supplementary high doses of Cr3 (50 and 500 mg/kg of diet, ca. 5 and 50 mg Cr/kg b.m.) given for 6 weeks did not affect tissular Fe levels in Fe-deficient and Fe-adequate healthy female rats.

Earlier studies [47–50] showed that chronic hyperglycaemia in diabetes could disorder the balance of some essential minerals (e.g. Fe, Zn, Cu, Mg) and thus worsen overall metabolic processes in the body.

The influence of diabetes on the Zn status has not been fully elucidated. In some animal trials [43] and clinical studies [51–53], decreased Zn levels were noticed in tissues and serum/plasma, respectively. The decreased Zn levels in diabetes may have been caused by disordered intestinal Zn absorption mechanisms and its increased urinary excretion. On the other hand, some other studies [27, 51] did not report significant changes in Zn status in the course of diabetes.

In our study, hyperglycaemia significantly decreased the renal Zn content, while supplementary Cr (both CrGly and CrPic) restored Zn levels. In our previous study [27], supplementary Cr3 (about 5 mg Cr/kg b.m.) normalized the liver Zn content in type 2 diabetic rats, but there were no changes in insulin-resistant model of rats [6, 45, 48].

Hyperglycaemia has been shown can also disturb Cu status [53–55]. Supplementary Cr3 in our previous trials, given at doses of 5 mg Cr/kg b.m. for 8 weeks, normalized the hepatic [6, 51], renal [27] and splenic Cu contents [6, 51, 52] in insulin-resistant and diabetic rats.

In our study, both compounds (CrGly and CrPic) normalized the serum, renal and cardiac molar Cu/Zn ratios and restored the kidney Zn and Cu contents. The lowering of the Cu/Zn molar ratio may be important due to the prevention of cardiovascular complications in diabetes. Since these two trace elements play an essential role in maintenance of redox balance (as cofactors of SOD), and hyperglycaemia per se affects Cu and Zn levels, disturbance in tissular proportion of Cu to Zn can further contribute to increasing free radical formation that are responsible for deterioration of tissues and organs as evidenced in diabetic complications [56, 57]. The production of reactive oxygen species (ROS) affects lipids. In diabetes, the level of lipid peroxidation markers such as malondialdehyde and thiobarbituric acid reactive substance (TBARS) increases [57]. Serum higher level of GST in type 2 diabetics was also observed [58]. There was positive relation between the CRP level and glycated hemoglobin observed in an earlier study on diabetics with poor glycaemia control (HbA1c > 9%) [59]. In this study, we only observed that the serum GST, TBARS and C-reactive protein tended to increase in diabetic rats. One of the weaknesses of this study is lack of tissular oxidative stress biomarkers (e.g. CAT, GPx and other lipid peroxidation indices) and inflammation (e.g. IL-6) that might shed more light on the degree of oxidative damage.

Another important aspect to be addressed in case of supplementary transition elements, here Cr, is the question of safety/toxicity. In this experiment, supranutritional dosages of Cr (7–8-fold Cr doses compared to the baseline) were used but did not affect liver enzymes (ALT, AST) values. Supplemented rats had the hepatic and renal Cr levels comparable with the values of the untreated groups that support previous observations that Cr3+ ions feature poor gastrointestinal absorption, fast urinary excretion and weak protein-binding affinity.

## Conclusions

Our study confirmed that the Cr(III) glycinate complex (GrGly) features appreciable hypoglycemic potential in the mild diabetic model of rats. Its effects were even more pronounced than those of chromium(III) picolinate. Apart from that, CrGly did not accumulate in the internal organs and it restored the balance of trace elements (Zn, Cu) in the diabetic rats.
